# Supplementary material for: Comparative Analysis of Serum and Serum-Free Medium Cultured Mesenchymal Stromal Cells for Cartilage Repair
Source: Int J Mol Sci. 2024 Oct 2;25(19):10627. doi: 10.3390/ijms251910627 (PMC11476526; doi:10.3390/ijms251910627)
Supplement: Supplementary file 1 [file ijms-25-10627-s001.zip › ijms-3208768-supplementary.pdf]

International Journal of
Molecular Sciences

MDPI

Supplementary Table S1. Modified O'Driscoll histological scoring system.

Category	Criteria	Score
	**1. Nature of Predominant Tissue**	
Cellular Morphology	Hyaline articular cartilage	4
	Incompletely differentiated mesenchyme	2
	Fibrous tissue or bone	0
Safranin-O Staining of the Matrix	Normal or nearly normal	3
	Moderate	2
	Slight	1
	None	0
Collagen Type 2 of Matrix	Normal or nearly normal	3
	Moderate	2
	Slight	1
	None	0
**2. Structural characteristics**		
Surface regularity	Smooth and intact	3
	Superficial horizontal lamination	2
	Fissures 25-100%	1
	Severe disruption including fibrillation	0
Structural integrity	Normal	2
	Slight disruption including cysts	1
	Severe disintegration	0
Thickness	100% of normal adjacent cartilage	2
	50-100% of normal cartilage	1
	0-50% of normal cartilage	0
Bonding to the adjacent cartilage	Bonded at both ends of graft	2
	Bonded at one end or partially at both ends	1
	Not bonded	0
**3. Freedom degeneration**		
Hypocellularity	normal cellularity	3
	Slight hypocellularity	2
	Moderate hypocellularity	1
	Severe hypocellularity	0
Chondrocyte Clustering	No clusters	2
	<25% of the cells	1
	25-100% of the cells	0
Freedom from degenerative changes in adjacent cartilage	Normal celluarity, no clusters, and normal staining	3
	Normal cellularity, mild clusters, and slight staining	2
	Mild or modeate hypocellularity, slight staining	1
	Server hypocelluarity, poor or no staining	0
